# Advancing the diagnosis of scrub typhus with targeted next-generation sequencing

**DOI:** 10.3389/fmicb.2026.1875741

**Published:** 2026-07-10

**Authors:** Weijuan Qin, Yuni Guo, Ming Lei, Huanhuan Wei, Zhengyi Liang, Liyan Zhou, Zhicong Li, Huimin Zhang, Xiaoning Wu, Li Xie

**Affiliations:** Department of Clinical Laboratory, The Second Affiliated Hospital of Guangxi Medical University, Nanning, Guangxi, China

**Keywords:** detection, diagnosis, *O. tsutsugamushi*, scrub typhus, TNGS, Weil–Felix test

## Abstract

**Objective:**

Scrub typhus has nonspecific symptoms, making it easy to miss or underdiagnose. This retrospective study evaluated targeted next-generation sequencing (tNGS) for the early diagnosis of acute *Orientia tsutsugamushi* infection in terms of feasibility and application value.

**Methods:**

This retrospective study included 49 patients with suspected scrub typhus as the case group. Among them, 28 patients with eschar or ulcers were assigned to the clinical diagnosis group. The remaining 21 patients without eschar or ulcers formed the clinically suspected group. Additionally, 48 patients with nonscrub typhus bloodstream infections during the same period were included in the negative control group. Clinical data and tNGS results were collected for all the patients.

**Results:**

No significant differences were found between the clinical diagnosis group and the clinically suspected group in sex, age, time from onset to sampling, season of onset, complications, or clinical prognosis (*p* > 0.05). The sensitivity of tNGS for diagnosing scrub typhus was 91.8% (45/49), and the specificity was 100.0% (48/48). No significant differences were observed between the clinically diagnosed group and the clinically suspected group regarding the positivity rate of *O. tsutsugamushi* detected by tNGS or the therapeutic response to doxycycline (*p* > 0.05). Significant changes were observed in the median eosinophil count (EOS), platelet count (PLT), aspartate aminotransferase (AST), and alanine aminotransferase (ALT) before and after doxycycline treatment in the case group (*p* < 0.001).

**Conclusion:**

TNGS demonstrated high sensitivity and specificity for the detection of *O. tsutsugamushi*. It facilitates the early diagnosis of scrub typhus, particularly in suspected cases without eschars. However, prospective studies are needed for validation.

## 1 Introduction

*Orientia tsutsugamushi* is an obligate intracellular bacterium that causes scrub typhus in humans through the bite of infected chigger mites. Clinical manifestations of scrub typhus include nonspecific fever, skin ulcers or eschars, thrombocytopenia, and liver function impairments, among others (Varghese et al., 2014). Because these symptoms are nonspecific and often resemble those of other locally endemic infections, scrub typhus is frequently missed and underdiagnosed (Marks et al., 2018; Mørch et al., 2017). Timely and accurate diagnosis, together with appropriate antimicrobial therapy, is essential for preventing complications and reducing mortality. The diagnosis of scrub typhus involves both clinical and laboratory methods. Clinical diagnosis primarily hinges on the presence of specific skin ulcers or eschars. However, diagnostically useful skin ulcers or eschars are present in only a subset of patients, with positivity rates varying across different countries and populations. Multiple meta-analyses have estimated that eschars are present in approximately 30% of cases (Dasgupta et al., 2024; Gupta et al., 2024; He et al., 2024). Therefore, most patients with *O. tsutsugamushi* infection require laboratory testing for accurate clinical diagnosis. Current laboratory approaches for diagnosing scrub typhus include the Weil–Felix test, detection of rising antibody titers or specific immunoglobulins (Ig) G or IgM using serological methods, and qPCR-based detection of *O. tsutsugamushi* nucleic acids. Although the Weil–Felix test is rapid and relatively easy to perform, its diagnostic value is limited by poor sensitivity and specificity (Lim et al., 2015; Wu et al., 2022). Serological testing remains the most commonly used approach for detecting *O. tsutsugamushi*, with the indirect immunofluorescence antibody (IFA) *assay* recognized as the gold standard serological test (Carroll and Pfaller, 2024; Peter et al., 2015). In patients with scrub typhus, specific antibodies typically do not become detectable until approximately 1 week after disease onset. As a result, serological testing is not suitable for the early diagnosis of scrub typhus. In some patients, antibody titers may continue to increase even after treatment has been completed (Gupta et al., 2016), and serology may also be positive in some healthy individuals (Wang et al., 2024). Therefore, relying solely on serological testing may result in missed diagnoses among patients who present early in the disease course. Compared with PCR, serological testing has lower sensitivity and specificity (Kim et al., 2021). PCR-based detection of *O. tsutsugamushi* has been widely reported, with real-time qPCR demonstrating high sensitivity and specificity (Kannan et al., 2020). However, the clinical manifestations of scrub typhus are nonspecific. Consequently, when the clinical suspicion of infection is unclear, clinicians may be less likely to consider testing for *O. tsutsugamushi*, which can readily lead to misdiagnosis or underdiagnosis. In contrast, tNGS does not require clinicians to identify a suspected pathogen because a single tNGS assay can simultaneously and broadly screen for more than 95% of clinically common bacteria, viruses, fungi, Rickettsia and other pathogens. Moreover, in recent years, multiple case reports have documented the successful diagnosis of scrub typhus using metagenomic next-generation sequencing (mNGS) (Basu and Chakravarty, 2022; Chen et al., 2026; Jian et al., 2024). However, only a few case reports have described the use of tNGS to support the early clinical diagnosis and treatment of scrub typhus (Chen et al., 2025; Gan et al., 2025; Lang et al., 2024; Wang et al., 2026). Therefore, this retrospective analysis was performed to assess the feasibility and clinical utility of tNGS for the early diagnosis of acute *O. tsutsugamushi* infection in the absence of convalescent-phase serum samples.

## 2 Materials and methods

### 2.1 Ethical statement

This study was approved by the Medical Ethics Committee of the Second Affiliated Hospital of Guangxi Medical University. Owing to the retrospective nature of the study and the absence of additional testing, the requirement for patient-informed consent was waived.

### 2.2 Subjects and grouping

This single-center retrospective study included 49 patients with clinically suspected scrub typhus admitted to the Second Affiliated Hospital of Guangxi Medical University from September 2023 to December 2025 as the case group. The inclusion criteria for the case group were as follows: (1) had a history of outdoor exposure within 3 weeks before disease onset, fever, lymphadenopathy, and/or rash; and (2) underwent complete tNGS testing on blood, bronchoalveolar lavage fluid, or sputum. The exclusion criterion was incomplete clinical data or the absence of tNGS testing. On the basis of these criteria and the availability of retrospective data, the case group was further stratified into a clinically diagnosed subgroup (*n* = 28), defined by the presence of a specific skin ulcer or eschar. Clinical Suspected Group (*n* = 21): Patients without specific skin ulcers or eschar. Concurrently, 48 hospitalized patients with bloodstream infections (BSIs) but without scrub typhus, who were diagnosed during the same period, were enrolled as the negative control group. The inclusion criteria were patients with fever and suspected bloodstream infection, with bacteria or fungi isolated from blood culture, and for whom blood tNGS did not detect *O. tsutsugamushi* but detected the same pathogen as the blood culture. The exclusion criteria were the same as those for the case group.

Data collection: Patient demographics; clinical manifestations; medication use; time to fever resolution; disease outcomes; and laboratory findings, including PLT, EOS, AST, ALT, tNGS and Weil–Felix test results. Qualitative real-time PCR (qPCR) was performed on 7 retained blood samples.

### 2.3 Targeted next-generation sequencing (tNGS)

The tNGS employed in this study was a targeted sequencing approach based on ultra-multiplex PCR amplification for library construction. Six primer pairs were used to detect *O. tsutsugamushi*, as detailed in Table 1. The use and implementation of tNGS followed expert consensus guidelines (The Laboratory Medicine Society, C. A. o. M. E, 2025). TNGS detection was performed by the Department of Medical Laboratory, Second Affiliated Hospital of Guangxi Medical University. A commercial assay kit was used for this procedure, with the target gene panel and primer sequences preoptimized by the manufacturer. For each sample, 500 μL of whole blood was placed in a 2 mL grinding tube and subjected to enzymatic lysis and bead-beating disruption. After cell lysis, DNA was extracted from the homogenate using a nucleic acid extraction or purification kit (Changsha Shengwei Digital Biotechnology Co., Ltd., China) following the manufacturer’s protocols. Reverse transcription was then performed using the following RT-PCR conditions: 25 °C for 10 min, 55 °C for 15 min, and 85 °C for 5 min. After reverse transcription, the RT-PCR products and nucleic acids isolated from whole-blood samples were mixed with the panel mix and 3X enzyme mix for the first round of PCR. The first-round PCR conditions consisted of initial denaturation at 95 °C for 3 min, followed by 23 cycles of denaturation at 95 °C for 20 s, annealing at 63 °C for 2 min, and extension at 72 °C for 2 min. Targeted PCR products obtained from the first round were subsequently purified using magnetic beads. The purified PCR products were subjected to a second round of amplification. The second-round PCR conditions consisted of initial denaturation at 95 °C for 3 min, followed by 12 cycles of denaturation at 95 °C for 15 s, annealing at 58 °C for 15 s, and extension at 72 °C for 1 min. The amplified products were then purified again using magnetic beads. Sequencing of the purified libraries was performed using a Universal Sequencing Reaction Kit (GeneMind Biosciences Co., Ltd., China).

**Table 1:** Primers used in the present study related to *O. tsutsugamushi.*

Target gene	Primer sequence (5′–3′)
*O. tsutsugamushi*, O113_07	F-CTTCAATAGAGGTACTAACGCCAGA
	R-AATAACGAGCTCCAGGTCTGACTTT
*O. tsutsugamushi*, O121_02	F-ACAGATTCTATAACACATTACAAATCTATTTT
	R-CATTTCGAACCTCAATTGTCGTAAA
*O. tsutsugamushi*, O121_02_s1	F-TAGTAGTAGATGAAGCTGGAATGGT
	R-TTCGAATATTTACTAAAACATGTGAACC
*O. tsutsugamushi*, O122_01_s1	F-ATGTTAGTGAGGAACAGAAACAAGC
	R-CAGCTCTGATACTGCCTTATGAGT
*O. tsutsugamushi*, O130_2	F-GCAGACATAGCAGGCATATAAAAAG
	R-TGTAGGTCTTAATCCTAAGCATCGT
*O. tsutsugamushi*, O130_2_s1	F-ACAAAAACAGATAAAGCAGACAGTG
	R-AAACATTCAGGCGATTAACTAGCT

### 2.4 Other testing methods for the case group

Weil–Felix test: Due to clinical practice variations, only 33 patients underwent the Weil–Felix test. The Weil–Felix test was performed using the *Proteus* OXK antigen. Serum samples were serially diluted with normal saline starting at a dilution of 1:40. An agglutination titer of ≥ 1:160 was considered positive.

qPCR: qPCR was performed on 7 retained blood samples from the case group using an *O. tsutsugamushi* detection kit (Shanghai Zhengke Biotechnology Co., Ltd., Shanghai, China). The reaction conditions and interpretation criteria were followed according to the kit instructions (a Ct value ≤40 was considered positive). Owing to the very small sample size, the results were not used for statistical comparisons.

### 2.5 Microbiological methods for the control group

Blood culture and tNGS test results of the control group were retrospectively collected. Blood culture and identification: In accordance with routine clinical procedures, BD blood culture bottles and a BD BACTEC blood culture system (BD Medical Devices (Shanghai) Co., Ltd., Shanghai, China) were used for culture. After a positive culture signal was detected, the samples were subcultured onto blood agar plates and incubated at 35 °C in a 5% CO₂ atmosphere for 16–24 h. The resulting colonies were selected and identified using a matrix-assisted laser desorption/ionization time-of-flight mass spectrometry (MALDI-TOF MS) system (Autof ms1000; Autobio Diagnostics Co., Ltd., Zhengzhou, China). TNGS was performed on blood samples from the control group using the same protocol as that used for the case group, which was conducted to evaluate the specificity of tNGS for detecting *O. tsutsugamushi*.

### 2.6 Statistical analysis

Continuous variables (age and time interval from symptom onset to sample collection) were tested for normality using the Shapiro–Wilk test. The data were normally distributed (*p* > 0.05) and are presented as the mean ± standard deviation (SD). The homogeneity of variance was assessed using Levene’s test. Since the variances were equal (*p* > 0.05), group comparisons were performed using the independent samples *t*-test. Categorical variables, including sex, season of onset, incidence of complications, clinical outcomes, tNGS positivity rate, and response to doxycycline therapy, are presented as frequencies and percentages (*n*(%)). Comparisons of categorical variables were performed using Pearson’s chi-square test for sex (all expected cell counts were ≥ 5) and Fisher’s exact test for the remaining variables. Continuous variables (PLT, EOS, AST, and ALT) were tested using the Shapiro–Wilk test, and all the variables were not normally distributed (*p* < 0.05). Data are presented as medians (interquartile ranges, *Q*1–*Q*3), and the Wilcoxon signed-rank test was used to compare the pretreatment and posttreatment values. All the statistical analyses were performed using SPSS version 27.0. All tests were two-tailed, and a *p*-value < 0.05 was considered to indicate statistical significance.

## 3 Results

### 3.1 Baseline clinical characteristics of the case group

The case group comprised 49 patients with clinically suspected scrub typhus, including 28 patients with a clinical diagnosis of scrub typhus (presence of specific skin ulcers or eschars) and 21 patients with clinical suspicion alone (absence of specific skin lesions). No significant differences were observed in demographic or clinical characteristics—including sex, age, or the time interval from onset to sampling—between the clinically diagnosed and clinically suspected subgroups. Cases occurred throughout spring to autumn, with peak concentrations in October and November. No significant difference was observed in the distribution of disease onset between the two subgroups (*p* > 0.05). Similarly, no significant differences were observed in the incidence of complications or clinical outcomes between the two subgroups. Notably, one patient in each subgroup developed intracranial infection. Additionally, one patient in the clinically suspected subgroup died because of intracranial hemorrhage, and another patient with active severe systemic lupus erythematosus (SLE) was discharged against medical advice without receiving targeted therapy. However, these sporadic events did not significantly alter the overall clinical outcomes between the two groups. Specific data are shown in Table 2 and Supplementary Table 1.

**Table 2:** Baseline clinical characteristics of the patients.

Variable	Clinically diagnosed group	Clinically suspected group	*p* value
Group definition	Presence of eschar	Absence of eschar	N/A
Sample size (*n*)	28	21	N/A
Gender % (*n*/*N*)			
Male	46.4 (13/28)	47.6 (10/21)	0.934
Female	53.6 (15/28)	52.4 (11/21)	
Age, years			
Mean ± SD	52.36 ± 18.30	61.19 ± 14.13	0.072
Interval from onset to sampling (*d*), Mean ± SD (Min–Max)	7.61 ± 2.90 (3–15)	7.29 ± 4.19 (1–15)	0.752
Season of onset % (*n*/*N*)			
Spring (March–May)	10.7 (3/28)	4.8 (1/21)	0.451
Summer (June–August)	25.0 (7/28)	42.9 (9/21)	
Autumn (September–November)	64.3 (18/28)	52.4 (11/21)	
Complications % (*n*/*N*)			
Hepatic dysfunction	92.9 (26/28)	90.5 (19/21)	1.000
Pulmonary infection	60.7 (17/28)	61.9 (13/21)	
Sepsis	35.7 (10/28)	33.3 (7/21)	
Renal dysfunction	32.1 (9/28)	33.3 (7/21)	
Cardiac dysfunction	32.1 (9/28)	33.3 (7/21)	
Outcomes % (*n*/*N*)			
Full recovery	100.0 (28/28)	90.5 (19/21)	0.417
Death	0.0 (0/28)	4.8 (1/21)	

N/A, Not applicable.

### 3.2 Diagnostic performance of tNGS for detecting *O. tsutsugamushi*

A total of 49 patients were included in the case group, 48 patients were included in the negative control group, and both groups underwent tNGS testing. In the case group, 45 patients tested positive for *O. tsutsugamushi,* and 4 tested negative, yielding a positivity rate of 91.8%. All 48 patients in the negative control group tested negative for *O. tsutsugamushi* by tNGS. On the basis of these results, the sensitivity and specificity of tNGS were 91.8% (45/49) and 100.0% (48/48), respectively. The positive predictive value (PPV) and negative predictive value (NPV) were 100.0% (45/45) and 92.3% (48/52), respectively. Details are shown in Tables 3 and 4, and detailed information on the patients in the negative control group is available in Supplementary Table 2.

**Table 3:** Detection results of *O. tsutsugamushi* by tNGS.

Detection result	Case group (*n*)	Control group (*n*)
tNGS positive (*O. tsutsugamushi*)	45	0
tNGS negative (*O. tsutsugamushi*)	4	48
Total	49	48

**Table 4:** Diagnostic performance of tNGS for detecting *O. tsutsugamushi*.

Indicator	Results
Sensitivity %	91.8%
Specificity %	100.0%
PPV %	100.0%
NPV %	92.3%

### 3.3 Comparison of tNGS detection rates and doxycycline treatment responses between the clinically diagnosed subgroup and the clinically suspected subgroup

The positivity rate of *O. tsutsugamushi* by tNGS was 85.7% (24/28) in the clinically diagnosed subgroup and 100% (21/21) in the clinically suspected subgroup. There was no statistically significant difference between the two groups (*p* = 0.125). In the clinically diagnosed group, 27 patients received doxycycline treatment. Among them, 19 patients (70.4%) achieved defervescence within 48 h. In the clinically suspected group, 18 patients received doxycycline treatment, and 15 patients (83.3%) achieved defervescence within 48 h. No statistically significant difference was observed in the doxycycline treatment response between the two subgroups (*p* = 0.482). The detailed data are shown in Table 5.

**Table 5:** TNGS results and doxycycline response in clinically diagnosed versus suspected cases.

Group	tNGS results		Response to doxycycline		
	Tested cases (*N*)	Positive rate % (*n*/*N*)	Treatment cases (*N*)	Fever resolution ≤ 48 h (%) (*n*/*N*)	Fever resolution > 48 h (%) (*n*/*N*)
Clinically diagnosed group	28	85.7 (24/28)	27	70.4 (19/27)	29.6 (8/27)
Clinically suspected group	21	100.0 (21/21)	18	83.3 (15/18)	16.7 (3/18)
*p* value	N/A	0.125	N/A	0.482	N/A

N/A, Not applicable.

### 3.4 Routine laboratory findings

Routine laboratory data were analyzed for 45 patients in the case group who received doxycycline treatment (27 in the clinically diagnosed group and 18 in the clinically suspected group). Significant improvements in laboratory parameters were observed after treatment. Specifically, the median PLT increased from 112 to 232 (*p* < 0.001), the EOS increased from 0.000 to 0.060 (*p* < 0.001), the AST decreased from 80 to 39 (*p* < 0.001), and the ALT level decreased from 65 to 43 (*p* < 0.001). Specific data are shown in Table 6 and Supplementary Table 3.

**Table 6:** Comparison of laboratory parameters before and after treatment.

Laboratory parameters	Median	25th percentile	75th percentile	*p*
Eos-Pre-treatmenta(×10⁹/L)	0.000	0.000	0.010	<0.001
Eos-Post-treatmenta(×10⁹/L)	0.060	0.024	0.142	
PLT-Pre-treatmenta(×10⁹/L)	112	77.0	185.5	<0.001
PLT-Post-treatmenta(×10⁹/L)	232	168.0	322.3	
AST-Pre-treatmenta(U/L)	80	56.5	115.5	<0.001
AST-Post-treatmenta(U/L)	39	31.0	58.0	
ALT-Pre-treatmenta(U/L)	65	49.0	108.0	<0.001
ALT-Post-treatmenta(U/L)	43	29.0	70.0	

### 3.5 Other laboratory findings

A total of 33 patients in the case group underwent the Weil–Felix test, including 20 in the clinically diagnosed group and 13 in the clinically suspected group. All patients had OXK titers < 1:160 and were negative. Owing to sample availability constraints, qPCR validation was completed for only 7 patients (4 from the clinically diagnosed subgroup and 3 from the clinically suspected subgroup). Among these patients, 5 tested positive for *O. tsutsugamushi* by qPCR. Owing to the very limited sample size, a consistency analysis between the qPCR and tNGS results was not performed. The raw qPCR data are provided in Supplementary Table 4.

## 4 Discussion

*Orientia tsutsugamushi* freely resides in the cytoplasm of trombiculid mites, also known as chiggers, and is transmitted transovarially. Humans acquire infection through the bite of infected chigger larvae, resulting in scrub typhus, also known as tsutsugamushi disease. Scrub typhus is likely among the most underrecognized, neglected, and severe but readily treatable diseases worldwide (Walker, 2016). Early diagnosis and timely initiation of targeted therapy can significantly increase cure rates (Chaudhry et al., 2019).

In this study, among 49 patients with suspected scrub typhus who underwent tNGS testing, the positivity rate for *O. tsutsugamushi* was 91.8% (45/49), whereas all 48 patients in the negative control group tested negative for *O. tsutsugamushi* by tNGS. The sensitivity of tNGS for diagnosing scrub typhus was 91.8% (45/49), and the specificity was 100.0% (48/48). There was no significant difference in the tNGS positivity rate for *O. tsutsugamushi* or the response to doxycycline treatment between the clinically diagnosed group with eschars and the clinically suspected group without eschars. There were significant differences in the median values of PLT, EOS, AST, and ALT before and after doxycycline treatment in the case group.

There was no significant difference in the seasonal distribution between the clinically diagnosed and suspected subgroups. The incidence peaked in October and November, which is consistent with previous reports (Han et al., 2024). Liver dysfunction was the most common complication in both the clinically diagnosed group and the suspected group, followed by pneumonia and sepsis. No significant differences were observed in the incidence rates of these complications between the two groups. The high incidence of liver dysfunction and pulmonary involvement observed in this cohort aligns well with previous reports (Chaturvedi et al., 2025; Paskey et al., 2024; Sriwongpan et al., 2013). Six patients presented with sepsis and did not become afebrile within 48 h after doxycycline treatment, which is consistent with previous reports (Regan et al., 2015). In certain regions of India, the incidence of acute kidney injury (AKI) in patients with scrub typhus has been reported to be as high as 53% (Kumar et al., 2014; Thakur et al., 2020). In contrast, the incidence of renal dysfunction was comparable between the two subgroups in our cohort and notably lower than that reported above. Mounting evidence suggests that scrub typhus can adversely affect both the central and peripheral nervous systems, potentially leading to fatal outcomes (Basu and Chakravarty, 2022; Chaturvedi et al., 2025; Khan et al., 2017). In this cohort, one patient from each subgroup presented with symptoms of central nervous system infection. Both patients showed complete resolution of symptoms following anti-scrub typhus therapy, suggesting that scrub typhus can involve the nervous system, which is consistent with the literature.

TNGS demonstrated exceptionally high specificity for detecting *O. tsutsugamushi*, with no false positives observed in the 48 negative controls, indicating that this assay exhibits no cross-reactivity. The sensitivity of tNGS for detecting *O. tsutsugamushi* was 91.84% (45/49). Notably, all four false-negative results were derived from patients in the clinically diagnosed subgroup with typical eschars, suggesting that a negative tNGS result does not rule out infection. Whether tNGS positivity is correlated with the interval from onset to sampling remains to be determined because of the limited sample size. However, no significant difference was observed in this interval between the clinically diagnosed group and the suspected group (*p* > 0.05). False-negative findings may be attributable to several factors, including a narrow window period of pathogenemia, pre-testing exposure to effective antibiotics, or a low abundance of the pathogen in blood samples. Clinical experience suggests that the diagnostic window for detecting *O. tsutsugamushi* by PCR in blood is relatively short, and the optimal timing for detection is correlated with the initial infectious dose (Jiang et al., 2003; Paris et al., 2015; Singhsilarak et al., 2005). In this study, the time interval between fever onset and sample collection ranged from 1 to 15 days, and tNGS successfully detected *O. tsutsugamushi* in patients across this entire interval. Thus, we did not observe an obvious window period restriction for tNGS within the first 15 days of illness. In the present study, although no difference in the sampling interval was observed between the two groups, false negatives still occurred, suggesting that factors other than sampling timing, such as antibiotic exposure or host factors, may play a more important role. Nevertheless, we recommend that clinicians collect blood samples for tNGS testing as early as possible during the disease course (e.g., within the first week of onset) to improve the detection rate.

In this cohort, the clinically suspected subgroup (lacking eschars or ulcers) accounted for 42.9% (21/49) of the cases. The tNGS positivity rate in this group was not significantly different from that in the clinically diagnosed group (*p* > 0.05), and the two groups also showed no significant difference in doxycycline treatment response, which was consistent between them. These findings indicate that tNGS is highly effective for detecting *O. tsutsugamushi* even in the absence of characteristic skin lesions. The prevalence of pathognomonic eschar or ulcers, key clinical signs of scrub typhus, varies considerably among different geographic regions and patient populations (Dasgupta et al., 2024; Gupta et al., 2024; He et al., 2024). In the present study, three patients did not exhibit any skin ulcers or eschars at the time of hospital admission; however, these lesions became evident upon discharge. Moreover, previous studies have identified the absence of an eschar as an independent predictor of fatal outcomes in scrub typhus patients (Lee et al., 2009). Therefore, for suspected scrub typhus patients without eschar, greater reliance on laboratory diagnosis is warranted. TNGS obviates the need for clinicians to pre-select specific pathogens, providing a reliable basis for early diagnosis in such atypical cases. This capability helps reduce missed diagnoses and treatment delays. In this study, tNGS utilized multiplex PCR to selectively enrich target pathogen fragments, thereby effectively mitigating interference from the human genomic background, significantly increasing the sensitivity of pathogen detection and enabling the simultaneous detection of dozens to hundreds of pathogens from a single sample. Compared with mNGS, tNGS offers advantages such as lower costs, shorter turnaround times, broader pathogen coverage, and superior sensitivity for target detection (National Clinical Research Center for Infectious Diseases, Infectious Diseases Professional Committee of Shenzhen Medical Association, Shenzhen Infectious Disease Quality Control Center, 2024; Society of Clinical Microbiology of China International Exchange and Promotion Association for Medical and Healthcare, 2024; The Laboratory Medicine Society, C. A. o. M. E, 2025), rendering it particularly suitable for patients with febrile illness of unknown origin or unclear infectious etiology. Recently, reports have described the successful diagnosis of scrub typhus using mNGS (Basu and Chakravarty, 2022; Chen et al., 2026; Jian et al., 2024). In contrast, reports on the application of tNGS in this field are scarce; the present study, therefore, offers an important addition to the literature.

A total of 45 patients from both the clinical diagnosis group and the suspected group were treated with doxycycline. The rates of defervescence within 48 h were high in both groups, and the difference was not statistically significant (*p* > 0.05), which is consistent with the therapeutic diagnostic criteria for doxycycline (Biggs et al., 2016). Following doxycycline therapy, PLT and EOS counts increased markedly, whereas AST and ALT levels decreased significantly (*p* < 0.001). These trends indicate the recovery of the hematopoietic system and liver function following pathogen clearance, which aligns with previous reports in the literature (Paskey et al., 2024; Sriwongpan et al., 2013). These therapeutic outcomes validate the reliability of tNGS from a clinical perspective; moreover, the comparable treatment responses between the two subgroups further indicate that tNGS can effectively guide targeted therapy in atypical cases.

In the case group, all 33 patients who underwent the Weil–Felix test yielded consistently negative results, whereas tNGS successfully identified 45 positive cases of *O.* tsutsugamushi in the same cohort, which further confirms that the Weil–Felix test is unsuitable for the early diagnosis of scrub typhus because of its low sensitivity, which is consistent with reports in the literature (Chaturvedi et al., 2025; Kannan et al., 2020; Lim et al., 2015). With respect to qPCR, this study was limited to only seven preserved samples available for analysis. Owing to this extremely limited sample size, a formal concordance analysis between qPCR and tNGS was not performed; thus, no reliable conclusions can be made regarding the comparison of the two methods. Nonetheless, the data from this cohort demonstrate a clear advantage of tNGS in the early diagnosis of scrub typhus. Particularly in cases where the Weil–Felix test is negative despite high clinical suspicion, tNGS offers a robust etiological basis for confirming the infection.

This study had a single-center, retrospective design. The case group was selected on the basis of clinical criteria, whereas the negative control group consisted of patients with confirmed bloodstream infections other than scrub typhus. This design may have led to an overestimation of the diagnostic efficacy of tNGS. Furthermore, the relatively small sample size (49 cases and 48 controls) introduces the potential for substantial sampling error, which may limit the stability and generalizability of the point estimates for sensitivity and specificity. Moreover, the Weil–Felix test was performed in only a subset of patients, and the qPCR validation was limited by a small sample size. Crucially, tNGS results were not directly compared against a gold standard, such as IFA or ELISA. The retrospective design and lack of retained samples (only 7 available) prevented any additional serological testing beyond the clinically performed Weil–Felix test, which is known for its poor sensitivity. Therefore, these preliminary findings require further validation through large-scale, multicenter, prospective studies.

In summary, our findings indicate that tNGS is highly sensitive and specific for the detection of *O. tsutsugamushi* and remains effective in suspected cases lacking eschar or ulcers, and the test results can inform targeted doxycycline therapy. In the absence of convalescent-phase serum samples, tNGS can serve as an effective tool for the early diagnosis of scrub typhus, particularly in patients with fever of unknown origin and a history of outdoor exposure but lacking characteristic signs. This study provides important evidence for the application of tNGS in the early diagnosis of scrub typhus; however, the results should be interpreted with caution, and prospective studies are warranted for validation.

## Data Availability

All data supporting the findings of this study are included in the manuscript and its supplementary tables.
