# Supplementary material for: Advancing the diagnosis of scrub typhus with targeted next-generation sequencing
Source: Front Microbiol. 2026 Jul 10;17:1875741. doi: 10.3389/fmicb.2026.1875741 (PMC13395850; doi:10.3389/fmicb.2026.1875741)
Supplement: Supplementary file 1 [file Data_Sheet_1.pdf]

SUPPLEMENTAL MATERIALS

Manuscript Title:

Advancing the Diagnosis of Scrub Typhus With Targeted Next-Generation Sequencing

Authors:

Weijuan Qin[1†], Yuni Guo[1†],Ming Lei[1], Huanhuan Wei[1], Zhengyi Liang[1], Liyan Zhou[1], Zhicong Li[1], Huimin Zhang[1], Xiaoning Wu[1*], Li Xie[1*]

Affiliations:

Department of Clinical Laboratory, The Second Affiliated Hospital of Guangxi Medical University, Nanning, Guangxi, China

Co-first Author：

Weijuan Qin, Yuni Guo

Co-corresponding Authors:

Li Xie, E-mail: drlixie@163.com;

Xiaoning Wu, E-mail: wxn535@163.com

Supplementary Table 1. Clinical features of patients with *O. tsutsugamushi*

Patient	Gender	Age, year	Month of presentation	Fever	Field exposure	Eschar	Hepatic Injury	Renal Injury	Cardiac Dysfunction	Pneumonia	Sepsis	Sampling interval[a] (d)	tNGS Results			Weil-Felix test
													Pathogen	Read Count	Pathogen Load(×10^3 Copies/mL)	
Patient 1	Male	61-65	Sep – Nov	Y	Y	Y	Y	Y	N	Y	Y	7	*O. tsutsugamushi*	87229	1000	Negative
Patient 2	Male	36-40	Sep – Nov	Y	Y	Y	Y	N	N	Y	Y	3	*O. tsutsugamushi*	88726	1000	Untested
Patient 3	Male	46-50	Sep – Nov	Y	Y	N	N	Y	N	Y	N	1	*O. tsutsugamushi*	97	0.4	Untested
Patient 4	Female	76-80	Sep – Nov	Y	Y	N	Y	Y	Y	N	N	6	*O. tsutsugamushi*	7237	2000	Negative
Patient 5	Female	56-60	Mar – May	Y	Y	Y	Y	Y	N	Y	N	4	*O. tsutsugamushi*	8459	3	Negative
Patient 6	Female	61-65	Mar – May	Y	Y	Y	Y	N	Y	Y	N	9	*O. tsutsugamushi*	337841	2000	Negative
Patient 7	Male	66-70	Mar – May	Y	Y	N	Y	N	N	Y	N	2	*O. tsutsugamushi*	370	8	Negative
Patient 8	Male	15-20	Mar – May	Y	Y	Y	Y	N	N	N	N	7	*O. tsutsugamushi*	154187	100	Negative
Patient 9	Male	51-55	Jun – Aug	Y	Y	Y	Y	N	Y	Y	Y	5	*O. tsutsugamushi*	197583	300	Negative
Patient 10	Female	66-70	Jun – Aug	Y	Y	Y[b]	Y	N	N	N	N	11	*O. tsutsugamushi*	124188	100	Negative
Patient 11	Female	66-70	Jun – Aug	Y	Y	N	Y	N	N	Y	Y	15	*O. tsutsugamushi*	9042	4	Negative
Patient 12	Female	61-65	Jun – Aug	Y	Y	N	Y	N	N	Y	N	11	*O. tsutsugamushi*	2016	0.8	Negative
Patient 13	Female	56-60	Sep – Nov	Y	Y	N	Y	Y	N	N	N	5	*O. tsutsugamushi*	9173	3	Negative
Patient 14	Male	66-70	Sep – Nov	Y	Y	Y	Y	N	N	Y	N	10	*O. tsutsugamushi*	10944	4	Negative

Patient 15	Female	76-80	Sep – Nov	Y	Y	Y	Y	N	N	Y	N	12	*O. tsutsugamushi*	380	0.1	Untested
Patient 16	Male	36-40	Sep – Nov	Y	Y	N	Y	N	N	N	N	10	*O. tsutsugamushi*	2473	1	Negative
Patient 17	Male	51-55	Sep – Nov	Y	Y	N	Y	N	N	N	N	7	*O. tsutsugamushi*	315	0.2	Untested
Patient 18	Male	51-55	Sep – Nov	Y	Y	Y	Y	N	N	N	N	8	*O. tsutsugamushi*	91253	40	Negative
Patient 19	Female	71-75	Sep – Nov	Y	Y	N	Y	N	N	Y	Y	10	*O. tsutsugamushi*	169844	100	Negative
Patient 20	Female	41-45	Sep – Nov	Y	Y	N	Y	Y	N	Y	Y	8	*O. tsutsugamushi*	1405	0.6	Negative
Patient 21	Female	86-90	Sep – Nov	Y	Y	Y[b]	Y	N	Y	Y	Y	11	*O. tsutsugamushi*	207703	100	Negative
Patient 22	Female	51-55	Sep – Nov	Y	Y	Y	Y	Y	Y	Y	N	7	*O. tsutsugamushi*	35044	10	Negative
Patient 23	Female	66-70	Jun – Aug	Y	Y	N	Y	N	N	Y	N	15	*O. tsutsugamushi*	13864	20	Untested
Patient 24	Male	56-60	Jun – Aug	Y	Y	N	Y	Y	N	N	N	10	*O. tsutsugamushi*	1482	3	Negative
Patient 25	Female	51-55	Jun – Aug	Y	Y	N	Y	N	N	N	N	2	*O. tsutsugamushi*	294	0.5	Negative
Patient 26	Male	36-40	Sep – Nov	Y	Y	Y	N	Y	N	N	N	4	*O. tsutsugamushi*	493	0.9	Untested
Patient 27	Male	31-35	Jun – Aug	Y	Y	Y	Y	N	N	N	N	8	*O. tsutsugamushi*	2186	0.9	Untested
Patient 28	Female	66-70	Jun – Aug	Y	Y	N	Y	Y	Y	Y	Y	4	*O. tsutsugamushi*	88828	0.2	Untested
Patient 29	Male	51-55	Jun – Aug	Y	Y	N	Y	N	N	N	Y	6	*O. tsutsugamushi*	16028	6	Untested
Patient 30	Female	56-60	Jun – Aug	Y	Y	Y	Y	Y	N	N	N	10	*O. tsutsugamushi*	387	0.1	Untested
Patient 31	Male	51-55	Jun – Aug	Y	N	Y	Y	N	N	N	N	4	*O. tsutsugamushi*	10891	4	Untested
Patient 32	Female	66-70	Jun – Aug	Y	Y	N	Y	N	Y	Y	Y	8	*O. tsutsugamushi*	1977	0.8	Negative
Patient 33	Male	81-85	Jun – Aug	Y	Y	N	Y	N	Y	Y	N	1	*O. tsutsugamushi*	5942	10	Untested
Patient 34	Male	31-35	Sep – Nov	Y	Y	N	N	N	N	N	N	6	*O. tsutsugamushi*	44728	20	Negative
Patient 35	Female	56-60	Jun – Aug	Y	Y	Y[b]	Y	N	N	N	N	8	*O. tsutsugamushi*	132	0.2	Negative
Patient 36	Female	46-50	Sep – Nov	Y	Y	Y	Y	N	N	N	N	7	Negative			Untested

Patient 37	Male	51-55	Sep – Nov	Y	Y	Y	Y	N	Y	Y	Y	7	Negative			Negative
Patient 38	Male	56-60	Jun – Aug	Y	Y	Y	Y	Y	N	Y	Y	5	Negative			Negative
Patient 39	Female	6-10	Sep – Nov	Y	Y	Y	Y	N	Y	Y	Y	9	*O. tsutsugamushi*	245	0.4	Untested
Patient 40	Female	21-25	Sep – Nov	Y	Y	Y	N	N	N	N	N	5	*O. tsutsugamushi*	86	0.1	Untested
Patient 41	Male	71-75	Sep – Nov	Y	Y	N	Y	Y	Y	Y	Y	13	*O. tsutsugamushi*	188081	300	Negative
Patient 42	Female	45-50	Sep – Nov	Y	Y	Y	Y	N	N	N	N	6	*O. tsutsugamushi*	10130	6	Negative
Patient 43	Male	45-50	Sep – Nov	Y	Y	Y	Y	Y	N	Y	Y	8	*O. tsutsugamushi*	8917	7	Negative
Patient 44	Female	86-90	Sep – Nov	Y	Y	Y	Y	Y	Y	Y	Y	5	*O. tsutsugamushi*	55633	100	Negative
Patient 45	Male	56-60	Sep – Nov	Y	Y	Y	Y	N	Y	Y	N	12	*O. tsutsugamushi*	472	0.8	Untested
Patient 46	Male	81-85	Sep – Nov	Y	Y	N	Y	N	Y	Y	N	5	*O. tsutsugamushi*	89	0.2	Negative
Patient 47	Female	36-40	Sep – Nov	Y	Y	Y	Y	Y	Y	Y	Y	6	*O. tsutsugamushi*	35321	50	Negative
Patient 48	Female	56-60	Sep – Nov	Y	Y	N	Y	N	Y	Y	N	8	*O. tsutsugamushi*	27643	20	Negative
Patient 49	Female	61-65	Sep – Nov	Y	Y	Y	Y	N	N	Y	N	15	Negative			Negative

a:Sampling interval refers to the time from symptom onset to collection of a specimen for tNGS testing; b:Black eschar appeared only just before discharge; Y: Yes, N: No.

Supplementary Table 2. Test results of the negative control group

**Control**	**Gender**	**Age，year**	**Blood Culture Result**	**tNGS Results**	
				**Read Count**	**Pathogen Load (×10^3 Copies/mL)**
Control 1	Female	46-50	*Salmonella*	155445	200
Control 2	Male	71-75	*Pseudomonas aeruginosa*	1226	0.3
Control 3	Female	76-80	*Pseudomonas aeruginosa*	2084	4

Control 4	Female	56-60	*Staphylococcus aureus*	95	0.2
Control 5	Male	41-45	*Staphylococcus aureus*	556	0.3
Control 6	Female	56-60	*Escherichia coli*	437	0.1
Control 7	Male	45-50	*Klebsiella pneumoniae*	464	0.8
Control 8	Female	71-75	*Candida albicans*	1245	2
Control 9	Female	71-75	*Escherichia coli*	616	0.6
Control 10	Male	61-65	*Staphylococcus epidermidis*	254	0.4
Control 11	Male	21-25	*Escherichia coli*	108	0.03
Control 12	Male	41-45	*Staphylococcus aureus*	185	0.3
Control 13	Male	51-55	*Escherichia coli*	73429	40
Control 14	Male	56-60	*Staphylococcus aureus*	197	0.4
Control 15	Female	76-80	*Escherichia coli*	118	0.03
Control 16	Male	56-60	*Candida albicans*	16	0.03
Control 17	Male	51-55	*Enterococcus faecalis*	10237	10
Control 18	Male	41-45	*Klebsiella pneumoniae*	3227	1
Control 19	Male	26-30	*Klebsiella pneumoniae*	427	0.8
Control 20	Male	41-45	*Escherichia coli*	161	0.3
Control 21	Female	66-70	*Enterococcus faecalis*	23118	20
Control 22	Male	31-35	*Staphylococcus aureus*	62	0.1
Control 23	Male	6-10	*Pseudomonas aeruginosa*	51	0.02
Control 24	Male	56-60	*Acinetobacter baumannii*	1389	0.9
Control 25	Female	81-85	*Escherichia coli*	2286	0.6

Control 26	Male	21-25	*Pseudomonas aeruginosa*	3234	0.7
Control 27	Female	51-55	*Escherichia coli*	8	0.007
Control 28	Female	36-40	*Escherichia coli*	78	0.005
Control 29	Male	56-60	*Klebsiella pneumoniae*	23358	40
Control 30	Female	66-70	*Candida albicans*	1721	3
Control 31	Male	46-50	*Escherichia coli*	2196	0.5
Control 32	Female	56-60	*Escherichia coli*	460	30
Control 33	Male	61-65	*Enterococcus faecalis*	8984	3
Control 34	Male	51-55	*Staphylococcus aureus*	5467	10
Control 35	Female	61-65	*Staphylococcus aureus*	123	0.2
Control 36	Male	56-60	*Pseudomonas aeruginosa*	83913	20
Control 37	Male	36-40	*Klebsiella pneumoniae*	77824	100
Control 38	Male	16-20	*Staphylococcus aureus*	4455	10
Control 39	Female	31-35	*Klebsiella pneumoniae*	707	0.2
Control 40	Female	71-75	*Candida parapsilosis*	3234	0.04
Control 41	Male	61-65	*Staphylococcus aureus*	15372	30
Control 42	Male	51-55	*Klebsiella pneumoniae*	71637	100
Control 43	Male	61-65	*Pseudomonas aeruginosa*	1541	0.3
Control 44	Male	26-30	*Escherichia coli*	3743	0.9
Control 45	Male	46-50	*Klebsiella pneumoniae*	94312	200
Control 46	Male	46-50	*Escherichia coli*	2099	0.5
Control 47	Male	36-40	*Staphylococcus aureus*	1252	0.8

Control 48	Male	36-40	*Pseudomonas aeruginosa*	4668	1

Supplementary Table 3. Laboratory results before and after doxycycline treatment

Patient	Time to Defervescence Post-Doxycycline (h)	Eos 0.02-0.52 x 10^9/L		PLT 125-350 x 10^9/L		AST（U/L） Adult: 15-40, Child: 13-35		ALT（U/L） Adult: 9-50，Child: 7-40	
		Pre-treatment[a]	Post-treatment[b]	Pre-treatment[a]	Post-treatment[b]	Pre-treatment[a]	Post-treatment[b]	Pre-treatment[a]	Post-treatment[b]
Patient 1	72	0.000	0.080	95	235	122	56	165	56
Patient 2	72	0.010	0.210	115	234	60	26	42	26
Patient 3	24	0.040	0.050	123	230	12	Untested	8	Untested
Patient 4	24	0.080	0.220	75	155	73	30	51	30
Patient 5	30	0.000	0.010	107	148	80	48	65	48
Patient 6	48	0.040	0.150	110	282	65	60	36	60
Patient 7	72	0.000	0.010	15	196	153	25	36	25
Patient 8	24	0.010	0.070	300	355	58	81	85	81
Patient 9	48	0.000	0.031	84	269	112	60	96	60
Patient 10	24	0.000	0.006	112	125	100	80	104	80
Patient 11	24	0.060	0.040	249	374	69	36	44	36
Patient 12	24	0.010	0.090	309	390	55	19	98	19
Patient 13	24	0.000	0.070	200	393	190	91	133	91
Patient 14	72	0.000	0.030	36	283	223	124	233	124
Patient 17	24	0.000	0.020	61	191	145	137	200	137

Patient 18	72	0.000	Untested	118	Untested	146	Untested	137	Untested
Patient 19	48	0.000	0.040	160	385	45	12	80	12
Patient 20	Persistent Fever, Death	0.000	Untested	45	Untested	90	Untested	79	Untested
Patient 21	72	0.000	0.120	62	56	119	17	52	17
Patient 22	24	0.030	0.480	184	509	46	19	51	19
Patient 23	12	0.000	0.020	67	97	75	29	55	29
Patient 24	24	0.000	0.060	91	206	109	51	64	51
Patient 25	24	0.010	0.750	173	266	84	30	70	30
Patient 26	24	0.000	0.060	98	169	20	14	44	14
Patient 27	24	0.000	0.180	187	208	68	126	117	126
Patient 30	24	0.010	0.220	138	195	102	64	103	64
Patient 31	>72	0.010	0.040	79	103	79	35	54	35
Patient 32	24	0.000	0.060	126	536	131	33	112	33
Patient 33	>72	0.010	Untested	120	Untested	126	Untested	58	Untested
Patient 34	48	0.000	0.000	102	126	36	42	44	42
Patient 35	48	0.000	0.070	256	308	36	Untested	71	Untested
Patient 36	48	0.020	0.070	213	353	310	208	444	208
Patient 37	48	0.010	0.025	30	438	80	79	114	79
Patient 38	48	0.000	0.010	173	138	92	54	63	54
Patient 39	>72	0.000	0.000	74	221	108	30	104	30
Patient 40	48	0.000	0.030	82	172	33	Untested	26	Untested
Patient 41	48	0.020	0.040	43	178	105	32	59	32
Patient 42	36	0.000	0.170	208	405	38	65	46	65
Patient 43	48	0.020	0.050	106	201	63	70	69	70

Patient 44	48	0.000	0.020	57	75	128	28	60	28
Patient 45	48	0.000	0.310	218	312	69	76	118	76
Patient 46	48	0.040	0.000	279	302	50	27	47	27
Patient 47	72	0.000	0.140	128	165	66	43	55	43
Patient 48	48	0.010	0.230	112	310	106	62	142	62
Patient 49	48	0.020	0.090	291	253	55	35	47	35

a: Before tigecycline treatment; b: After tigecycline treatment.

Supplementary Table 4. The results of tNGS and qPCR

Patient	tNGS Results			PCR		Eschar
	Pathogen	Read Count	Pathogen Load （x 10^3 Copies/mL)	CT value	Result	
Patient 41	*O. tsutsugamushi*	188081	300	31.54	Positive	Y
Patient 42	*O. tsutsugamushi*	10130	6	37.78	Positive	Y
Patient 44	*O. tsutsugamushi*	55633	100	31.36	Positive	Y
Patient 46	*O. tsutsugamushi*	89	0.2	Negative	Negative	N
Patient 47	*O. tsutsugamushi*	35321	50	30.9	Positive	Y
Patient 48	*O. tsutsugamushi*	27643	20	34.93	Positive	N
Patient 49	Negative			Negative	Negative	Y

Y:Yes, N: No.
